# Association of Antenatal Steroid Exposure at 21 to 22 Weeks of Gestation With Neonatal Survival and Survival Without Morbidities

**DOI:** 10.1001/jamanetworkopen.2022.33331

**Published:** 2022-09-26

**Authors:** Sanjay Chawla, Myra H. Wyckoff, Matthew A. Rysavy, Ravi Mangal Patel, Dhuly Chowdhury, Girija Natarajan, Abbot R. Laptook, Satyan Lakshminrusimha, Edward F. Bell, Seetha Shankaran, Krisa P. Van Meurs, Namasivayam Ambalavanan, Rachel G. Greenberg, Noelle Younge, Erika F. Werner, Abhik Das, Waldemar A. Carlo

**Affiliations:** 1 Departments of Pediatrics, Central Michigan University, Wayne State University, Children’s Hospital of Michigan, Detroit; 2 Department of Pediatrics, UT Southwestern Medical Center, Dallas, Texas; 3 Department of Pediatrics, McGovern Medical School, The University of Texas Health Science Center, Houston; 4 Department of Pediatrics, Emory University School of Medicine and Children’s Healthcare of Atlanta, Atlanta, Georgia; 5 RTI International, Rockville, Maryland; 6 Department of Pediatrics, Women and Infants Hospital, Providence, Rhode Island; 7 Department of Pediatrics, UC Davis Children’s Hospital, Sacramento, California; 8 Department of Pediatrics, University of Iowa, Iowa City; 9 Department of Pediatrics, Wayne State University, Detroit, Michigan; 10 Division of Neonatal and Developmental Medicine, Stanford University School of Medicine, Palo Alto, California; 11 Department of Pediatrics, University of Alabama at Birmingham, Birmingham; 12 Department of Pediatrics, Duke University, Durham, North Carolina; 13 Department of Obstetrics and Gynecology, Women and Infants Hospital, Alpert Medical School of Brown University, Providence, Rhode Island; 14 Social, Statistical, and Environmental Sciences Unit, RTI International, Rockville, Maryland

## Abstract

**Importance:**

The provision of antenatal corticosteroids to pregnant patients at gestational age (GA) 22 6/7 weeks or less remains controversial and lacks support from randomized clinical trials.

**Objective:**

To compare rates of survival and survival without major morbidities among infants born at GA 22 0/7 to 23 6/7 weeks after exposure to antenatal steroids at 22 6/7 weeks’ gestation or less vs no exposure to antenatal steroids.

**Design, Setting, and Participants:**

This cohort study enrolled infants born at GA 22 0/7 to 23 6/7 weeks between January 1, 2016, and December 31, 2019, at centers in the National Institute of Child Health and Human Development Neonatal Research Network. Infants who did not receive intensive care and infants with antenatal steroid exposure after GA 22 6/7 weeks were excluded.

**Exposure:**

Infants were classified as having no, partial, or complete exposure to antenatal steroids.

**Main Outcomes and Measures:**

The primary outcome was survival to discharge. The main secondary outcome was survival without major neonatal morbidity. The associations of differential exposures to antenatal steroids with outcomes were evaluated using logistic regression, adjusting for GA, sex, race, maternal education, small for GA status, mode of delivery, multiple birth, prolonged rupture of membranes, year of birth, and Neonatal Research Network center.

**Results:**

A total of 431 infants (mean [SD] GA, 22.6 [0.5] weeks; 232 [53.8%] boys) were included, with 110 infants (25.5%) receiving no antenatal steroids, 80 infants (18.6%) receiving partial antenatal steroids, and 241 infants (55.9%) receiving complete antenatal steroids. Seventeen infants were exposed to antenatal steroids at GA 21 weeks. Among infants exposed to complete antenatal steroids, 130 (53.9%) survived to discharge, compared with 30 infants (37.5%) with partial antenatal steroid exposure and 239 infants (35.5%) with no antenatal steroids. Infants born after complete antenatal steroid exposure, compared with those without antenatal steroid exposure, were more likely to survive to discharge (adjusted odds ratio [aOR], 1.95 [95% CI, 1.07-3.56]) and to survive without major morbidity (aOR, 2.74 [95% CI, 1.19-6.30]).

**Conclusions and Relevance:**

In this retrospective cohort study, among infants born between GA 22 0/7 and 23 6/7 weeks who received intensive care, exposure to a complete course of antenatal steroids at GA 22 6/7 weeks or less was independently associated with greater odds of survival and survival without major morbidity. These data suggest that the use of antenatal steroids in patients at GA 22 6/7 weeks or less could be beneficial when active treatment is considered.

## Key Points

**Question:**

What is the association of antenatal steroid administration at 22 weeks’ gestational age (GA) or earlier with survival and morbidities in extremely preterm neonates?

**Findings:**

In this cohort study among 431 infants born between GA 22 0/7 and 23 6/7 weeks who received intensive care, exposure to a complete course of antenatal steroids at GA 21 to 22 weeks was independently associated with higher survival, compared with partial antenatal corticosteroids and no antenatal steroids.

**Meaning:**

These findings suggest that antenatal steroids should be administered promptly even for patients at risk of delivery at 22 weeks of gestation when postnatal resuscitation is planned, as exposure to a complete course of antenatal steroids was associated with significant improvement in survival and decreased morbidity.

## Teaser

This cohort study assesses rates of survival and survival without major morbidities among infants born at gestational age 22 0/7 to 23 6/7 weeks after exposure to antenatal corticosteroids at 22 6/7 weeks’ gestation or less vs no exposure to antenatal steroids.

## Introduction

Administration of antenatal corticosteroids to pregnant patients at risk for delivery between 24 to 34 weeks’ gestation is associated with improved short-term outcomes in preterm infants, including lower risk of mortality, respiratory distress syndrome (RDS), necrotizing enterocolitis (NEC), and intracranial hemorrhage (ICH).^1,2,3^ Both the American College of Obstetricians and Gynecologists (ACOG) and Society for Maternal-Fetal Medicine (SMFM) recommend considering neonatal assessment for resuscitation at gestational age (GA) 22 and 23 weeks. A September 2021 policy update^4^ by the ACOG and SMFM suggests that antenatal steroids may be considered starting at GA 22 0/7 weeks if neonatal resuscitation is planned and after appropriate counseling (weak recommendation, low-quality evidence). Recent guidelines by the British Association of Perinatal Medicine also support the use of antenatal steroids at GA 22 weeks with favorable risk factors after discussion with parents.^5^ However, owing to a lack of robust evidence, guidelines for providing antenatal steroids at these early gestations vary around the world.

Fewer than 50 infants known to be younger than GA 26 weeks have been enrolled in clinical trials of antenatal steroids.^1^ To our knowledge, no randomized clinical trial has assessed the benefits of antenatal steroids administration at GA 24 weeks or younger.^1^ In observational studies, antenatal steroid exposure is associated with improved survival, neonatal morbidity, and early childhood neurodevelopmental outcomes for extremely preterm infants as a collective group (GA 22 weeks to 25 weeks).^6,7,8,9^ Individual observational studies have varied in reports of the potential benefits associated with antenatal steroid exposure at 22 weeks.^6,9,10,11^ It is important to separately consider the benefits of antenatal steroid exposure at the earliest gestations due to differences in physiology, including potential differences in fetal receptor function, volume of distribution, and organ function.^12^

For clinicians caring for pregnant patients at 22 weeks’ gestation at risk of preterm delivery, the value of antenatal steroids administration at GA 22 weeks or younger remains unclear. For this reason, the objective of this study was to compare rates of survival and survival without major neonatal morbidities among infants born at GA 22 0/7 to 23 6/7 weeks who were exposed to antenatal steroids at GA 22 6/7 weeks or younger vs infants who were not exposed to antenatal steroids.

## Methods

### Study Design

This was a retrospective cohort study using prospectively collected data from the extremely preterm infant registry of the Eunice Kennedy Shriver National Institute of Child Health and Human Development Neonatal Research Network (NRN). The NRN Generic Database Registry was approved by the institutional review board at each site, with waiver of consent granted at all except 3 sites, where written or oral parental consent was obtained. This study is reported following the Strengthening the Reporting of Observational Studies in Epidemiology (STROBE) reporting guideline.

### Study Population

All infants born from January 2016 to December 2019 at NRN sites at a GA of 22 0/7 through 23 6/7 weeks, as determined by best obstetric estimate (when available) or based on postnatal examination were eligible. Infants who were outborn, had major congenital anomalies, received antenatal dexamethasone, received antenatal steroids at GA 23 weeks, or received more than 1 course of antenatal steroids were excluded. Infants who died within 12 hours without receiving postnatal life support were also excluded. Postnatal life support was defined as receipt of any of the following: endotracheal intubation, surfactant therapy, continuous positive airway pressure, manual ventilation or mechanical ventilation, chest compressions, epinephrine, volume resuscitation, blood pressure support, or parenteral nutrition.^13^

### Study Cohorts and Data Collected

Infants were classified into 3 groups: no antenatal steroids group, partial antenatal steroids group (1 dose of betamethasone), and complete antenatal steroids group (2 doses of betamethasone). A complete course of antenatal steroids is defined as administration of 2 intramuscular doses of betamethasone given 24 hours apart or 4 doses of dexamethasone given 12 hours apart. As dexamethasone was rarely used, 18 patients who were exposed to dexamethasone were excluded. Maternal data, including race, gestational age by best obstetric estimate, marital status, maternal health insurance, hypertension, diabetes prior to pregnancy, gestational diabetes, acute histological chorioamnionitis, clinical chorioamnionitis, prolonged rupture of membranes (PROM; >18 hours), mode of delivery, multiple births, and antenatal steroids administration data were collected. Race was self-reported and categorized as Black, White, and other, including American Indian or Alaskan Native, Asian, Native Hawaiian or other Pacific Islander, more than one race, and unknown or not reported. Race was included because it is known to be associated with differences in care and outcomes in preterm infants. Neonatal data were collected until death, discharge, or 120 days, whichever occurred first. Neonatal data included birth weight, sex, Apgar scores, receipt of chest compressions or epinephrine in delivery room, severe ICH, cystic periventricular leukomalacia (CPVL), severe bronchopulmonary dysplasia (BPD), NEC, spontaneous intestinal perforation, RDS, patent ductus arteriosus, systemic steroids for BPD, culture-proven sepsis at any time prior to discharge, retinopathy of prematurity (ROP), treatment for ROP, postmenstrual age at discharge, length of hospital stay, number of days receiving mechanical ventilation, and mortality.

### Definitions of Neonatal Morbidities and Outcomes

Severe ICH was defined as grade 3 or 4 ICH,^14^ based on the most severe head ultrasonography findings prior to hospital discharge, transfer, or death. Resuscitation in the delivery room was defined as receipt of chest compression or epinephrine. NEC was defined as modified Bell stage IIA or greater.^15,16^ Severe (grade 3) BPD was defined as receipt of invasive mechanical ventilation at 36 weeks postmenstrual age.^17^ Severe ROP was defined as ROP receiving medical or surgical treatment.

The primary outcome was survival to hospital discharge. Secondary outcomes were survival without severe neonatal morbidities, including severe ICH, CPVL, surgical NEC, severe BPD, and severe ROP requiring treatment and composites of individual morbidities or death.

### Statistical Analysis

Continuous variables were described using median and IQR, and categorical variables were described using frequency and percentage. Outcome variables were compared among study groups (no, partial, and complete antenatal steroids) using χ^2^ test for categorical variables, analysis of variance for continuous normally distributed variables, and Kruskal-Wallis test for continuous skewed variables. Generalized linear mixed models were developed for primary and secondary outcomes, controlling for known factors associated with survival and morbidities, including race, sex, GA in weeks, mode of delivery, multiple gestation, PROM, SGA status, and birth year, as fixed effects and center of birth as a random effect. As previous studies have noted a dose-dependent association of antenatal steroids with neonatal morbidities and 2-year neurodevelopmental outcome,^6^ a separate analysis on outcome of infants with and without exposure to a complete course of antenatal steroids was performed. E-value^18^ for the primary outcome was calculated to quantify the potential impact of any unmeasured confounder. No imputation was performed for missing data. Two-sided *P* values are reported throughout, with *P* < .05 considered statistically significant. Statistical analyses were conducted using SAS software version 9.4 (SAS Institute). Data were analyzed from September 2020 to August 2022.

## Results

**Figure 1.**
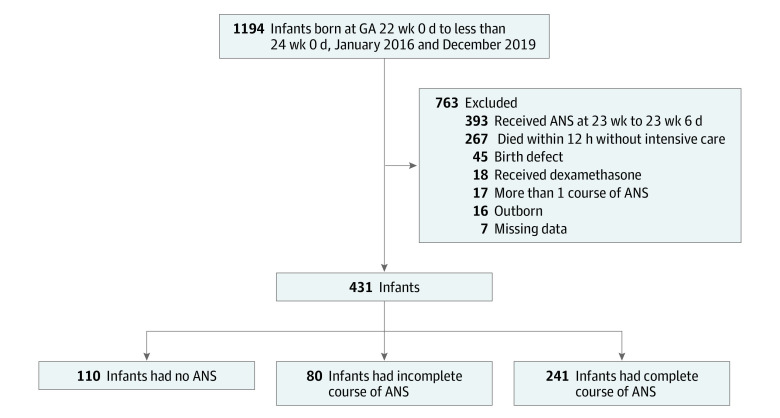
Flowchart of Participants During Study Period ANS indicates antenatal corticosteroids; GA, gestational age.

There were 1194 neonates born at NRN centers during the study period. Of these, 763 infants were excluded from this study (Figure 1). A total of 431 infants (mean [SD] GA, 22.6 [0.5] weeks; 232 [53.8%] boys) were included, with 110 infants (25.5%) receiving no antenatal steroids, 80 infants (18.6%) receiving partial antenatal steroids, and 241 infants (55.9%) receiving complete antenatal steroids. Differences in the maternal and infant characteristics were present among groups for maternal education, magnesium sulfate exposure, antibiotic exposure, clinical chorioamnionitis, histologic chorioamnionitis, PROM, mode of delivery, race, GA, Medicaid insurance, and Apgar scores at 1 and 5 minutes (Table 1). Seventeen infants were exposed to antenatal steroids at GA 21 weeks and 304 at 22 weeks. The earliest antenatal steroid exposure was at GA 21^2/7^ weeks.

**Table 1.** Maternal and Neonatal Characteristics by Exposure to ANS

Variable	No./No. with data (%)			*P* value^c^
	Complete (n = 241)^a^	Partial ANS (n = 80)^b^	No ANS exposure (n = 110)	
Married maternal status	109/241 (45.2)	27/80 (33.8)	48/108 (44.4)	.18
Maternal education >high school	132/210 (62.9)	30/65 (46.2)	39/95 (41.1)	<.001
Maternal magnesium sulfate exposure	196/241 (81.3)	48/80 (60.0)	14/110 (12.7)	<.001
Maternal antibiotic exposure	211/241 (87.6)	60/80 (75.0)	45/110 (40.9)	<.001
Hypertensive disorder of pregnancy	15/240 (6.3)	3/78 (3.8)	2/110 (1.8)	.18
Clinical chorioamnionitis	62/241 (25.7)	17/80 (21.3)	15/110 (13.6)	.04
Histological chorioamnionitis	185/235 (78.7)	58/71 (81.7)	57/104 (54.8)	<.001
Rupture of membrane >18 h	88/237 (37.1)	24/79 (30.4)	18/105 (17.1)	.01
Cesarean delivery	107/241 (44.4)	9/80 (11.3)	35/110 (31.8)	<.001
Singleton	156/241 (64.7)	56/80 (70.0)	79/110 (71.8)	.37
Race				
Black	110/237 (46.4)	49/77 (63.6)	44/102 (43.1)	.01
White	115/237 (48.5)	23/77 (29.9)	55/102 (53.9)	.01
Other^d^	12/237 (5.1)	5/77 (6.5)	3/102 (2.9)	.53
Infant sex				
Girls	107/241 (44.4)	42/80 (52.5)	50/110 (45.5)	.45
Boys	134/241 (55.6)	38/80 (47.5)	60/110 (54.5)	.45
GA at birth, wk				
22 wk	68/241 (28.2)	75/80 (93.8)	36/110 (32.7)	<.001
23 wk	173/241 (71.8)	5/80 (6.3)	74/110 (67.3)	<.001
Median (IQR), d	162 (160-164)	159 (157-160)	162 (159-164)	<.001
Median (IQR), wk	23 (22-23)	22 (22-22)	23 (22-23)	<.001
Birth weight, median (IQR) g	549 (494-605)	530 (472-581)	545 (495-620)	.24
SGA	11/240 (4.6)	0/80 (0.0)	6/110 (5.5)	.13
Medicaid insurance	115/241 (47.7)	50/79 (63.3)	71/109 (65.1)	.01
Apgar score, median (IQR)				
1 min	2 (1-4)	1 (1-3)	1 (1-2)	<.001
5 min	5 (3-7)	4 (1-6)	3 (2-5)	<.001
Resuscitation in the delivery room				
Chest compression	9/240 (3.8)	4/79 (5.1)	7/110 (6.4)	.55
Epinephrine	7/240 (2.9)	4/79 (5.1)	6/110 (5.5)	.45

Abbreviations: ANS, antenatal steroids; GA, gestational age; SGA, small for gestational age.

^a^ Complete course of ANS was defined as 2 doses of betamethasone at GA 22 6/7 or earlier.

^b^ Partial course of ANS was defined as 1 dose of betamethasone at GA 22 6/7 or earlier.

^c^ *P* value refers to overall significance among the three groups without any adjustment for covariates.

^d^ Races included in other category were American Indian or Alaskan Native, Asian, Native Hawaiian or Other Pacific Islander, more than one race, and unknown or not reported race.

**Figure 2.**
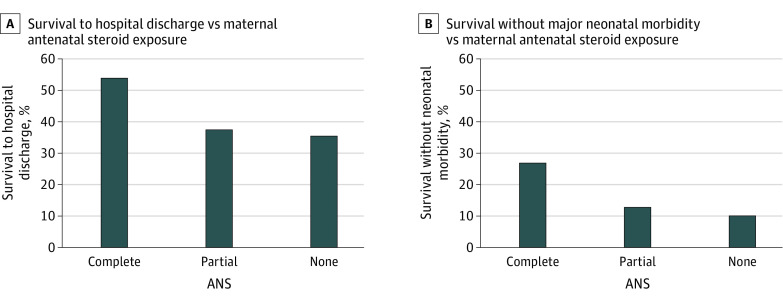
Survival to Hospital Discharge and Survival Without Major Neonatal Morbidity in Relation to Maternal Antenatal Steroid (ANS) Exposure

In adjusted analysis, there was a difference among antenatal steroid exposure groups in survival to hospital discharge and survival without major morbidity (Table 2). Among infants exposed to complete antenatal steroids, 130 (53.9%) survived to discharge, compared with 30 infants (37.5%) with partial antenatal steroids and 239 infants (35.5%) with no antenatal steroids (Figure 2). Among infants exposed to complete antenatal steroids, 64 (26.9%) survived without major morbidities, compared with 10 infants (12.8%) with partial antenatal steroids and 11 infants (10.0%) with no antenatal steroids (Figure 2). Compared with infants without antenatal steroid exposure, infants born after complete antenatal steroid exposure had significantly higher adjusted odds of survival to discharge (adjusted odds ratio [aOR], 1.95 [95% CI, 1.07-3.56]) and of survival without major morbidity (aOR, 2.74 [95% CI, 1.19-6.30]) (Table 2). When looking at individual morbidities, the risk of sepsis was higher in infants born after complete antenatal steroid exposure compared with those with partial antenatal steroid exposure (Table 2). The neonatal outcome of death, survival without any morbidity, and survival with number of severe morbidities (severe ICH, CPVL, surgical NEC, severe BPD, severe ROP needing treatment) stratified by antenatal steroid exposure is noted in the eFigure in Supplement 1.

**Table 2.** Neonatal Survival and Morbidities Among Infants Born After a Complete Course, Partial Course, or No Exposure to ANS

Variable	No./No. with data (%) with data			Adjusted *P* values	Adjusted odds ratio (95% CI)^a^		
	Complete ANS (n = 241)^b^	Partial ANS (n = 80)^c^	No ANS exposure (n = 110)		Partial vs no ANS	Complete vs no ANS	Complete vs partial ANS
Survival at hospital discharge	130/241 (53.9)	30/80 (37.5)	39/110 (35.5)	.08	1.41 (0.64-3.09)	1.95 (1.07-3.56)	1.39 (0.71-2.72)
Survival at 36 wk PMA	137/241 (56.8)	31/80 (38.8)	41/110 (37.3)	.04	1.43 (0.65-3.12)	2.11 (1.16-3.84)	1.48 (0.76-2.89)
Survival at 36 wk PMA without major neonatal morbidities^d^	64/238 (26.9)	10/78 (12.8)	11/110 (10.0)	.04	1.63 (0.53-5.00)	2.74 (1.19-6.30)	1.68 (0.69-4.09)
Severe ICH	63/199 (31.7)	26/55 (47.3)	34/65 (52.3)	.03	0.83 (0.31-2.17)	0.41 (0.20-0.85)	0.49 (0.22-1.11)
Severe ICH or death^e^	135/236 (57.2)	58/77 (75.3)	88/110 (80.0)	.01	0.54 (0.21-1.36)	0.33 (0.16-0.67)	0.62 (0.29-1.32)
CPVL	11/199 (5.5)	2/55 (3.6)	9/65 (13.8)	.29	0.45 (0.06-3.21)	0.41 (0.13-1.26)	0.91 (0.15-5.62)
CPVL or death^e^	112/236 (47.5)	51/77 (66.2)	75/110 (68.2)	.03	0.71 (0.32-1.61)	0.45 (0.24-0.82)	0.62 (0.31-1.26)
Grade 3 BPD	22/130 (16.9)	7/29 (24.1)	7/41 (17.1)	.98	1.18 (0.24-5.68)	1.09 (0.37-3.25)	0.93 (0.26-3.33)
Grade 3 BPD or death^e^	126/234 (53.8)	56/78 (71.8)	76/110 (69.1)	.16	0.73 (0.32-1.65)	0.56 (0.30-1.02)	0.77 (0.38-1.55)
Severe ROP needing treatment	41/140 (29.3)	8/31 (25.8)	18/41 (43.9)	.33	0.39 (0.09-1.67)	0.50 (0.18-1.39)	1.28 (0.37-4.42)
Severe ROP needing treatment or death^e^	148/239 (61.9)	58/78 (74.4)	88/110 (80.0)	.07	0.51 (0.20-1.33)	0.43 (0.21-0.89)	0.84 (0.39-1.81)
NEC	26/212 (12.3)	6/61 (9.8)	5/72 (6.9)	.74	1.47 (0.34-6.39)	1.53 (0.52-4.48)	1.04 (0.32-3.42)
NEC or death^e^	124/237 (52.3)	52/77 (67.5)	72/110 (65.5)	.36	0.96 (0.43-2.14)	0.68 (0.37-1.24)	0.71 (0.35-1.43)
Surgical NEC	14/212 (6.6)	1/61 (1.6)	3/72 (4.2)	.38	0.28 (0.02-3.37)	1.38 (0.35-5.42)	4.91(0.50-47.91)
Surgical NEC or death^e^	113/237 (47.7)	49/77 (63.6)	70/110 (63.6)	.20	0.80 (0.36-1.77)	0.59 (0.32-1.07)	0.73 (0.37-1.46)
Patent ductus arteriosus	139/214 (65.0)	33/61 (54.1)	40/72 (55.6)	.51	0.76 (0.30-1.91)	1.18 (0.58-2.41)	1.56 (0.73-3.35)
PDA or death^e^	206/239 (86.2)	70/78 (89.7)	100/110 (90.9)	.35	0.51 (0.15-1.75)	0.50 (0.20-1.30)	0.99 (0.35-2.74)
Sepsis	100/199 (50.3)	23/53 (43.4)	25/61 (41.0)	.08	0.57 (0.23-1.45)	1.36 (0.69-2.66)	2.37 (1.09-5.18)
Sepsis or death^e^	160/237 (67.5)	59/77 (76.6)	87/110 (79.1)	.17	0.48 (0.19-1.23)	0.54 (0.27-1.07)	1.11 (0.51-2.45)
Late-onset sepsis	92/197 (46.7)	19/51 (37.3)	24/61 (39.3)	.07	0.51 (0.20-1.30)	1.28 (0.65-2.49)	2.52 (1.14-5.61)
Late-onset sepsis or death^e^	156/237 (65.8)	58/77 (75.3)	87/110 (79.1)	.11	0.46 (0.18-1.16)	0.50 (0.25-0.99)	1.08 (0.50-2.34)

Abbreviations: ANS, antenatal steroids; BPD, bronchopulmonary dysplasia; CPVL, cystic periventricular leukomalacia; GA, gestational age; ICH, intracranial hemorrhage; NEC, necrotizing enterocolitis; PDA, patent ductus arteriosus; PMA, postmenstrual age; ROP, retinopathy of prematurity.

^a^ Model adjusted for GA, sex, race, maternal education, small for gestation (SGA), mode of delivery, multiple birth, prolonged rupture of membranes, year and center of birth as a random effect. Models for CPVL, severe BPD, NEC, surgical NEC, sepsis, and late-onset sepsis could not adjust for center of birth due to small sample size.

^b^ Complete course of ANS was defined as 2 doses of betamethasone at GA 22 6/7 or earlier.

^c^ Partial course of ANS was defined as 1 dose of betamethasone at GA 22 6/7 or earlier.

^d^ Presence of either severe ICH, cystic PVL, severe BPD, surgical NEC, or severe ROP requiring treatment.

^e^ Composite outcomes include death before GA 36 weeks for BPD, NEC, PDA, sepsis, late-onset sepsis and IVH/PVL, and death before discharge for severe ROP.

The E-value for survival at hospital discharge was 3.56 (lower confidence limit, 1.34) for complete vs no antenatal steroids. Thus, for the complete vs no antenatal steroids comparison, any unmeasured confounder associated with both survival at hospital discharge and antenatal steroids by an odds ratio of 3.56 could explain away the observed association between antenatal steroids and survival at hospital discharge, but weaker confounding would not.

In the analysis of dose response, the adjusted odds of survival at discharge and survival without major morbidity were significantly higher in infants born after complete antenatal steroid exposure compared with those without any antenatal steroids or with partial antenatal steroids (Table 3). The adjusted odds of severe ICH, severe ICH or death, and CPVL or death were significantly lower in infants born after complete antenatal steroid exposure compared to those without any antenatal steroids or partial antenatal steroid exposure.

**Table 3.** Neonatal Survival and Survival Without Morbidity of Infants Born With and Without Exposure to a Complete Course of ANS

Variable	No./No. (%)		Complete vs partial or no ANS, adjusted OR (95% CI)^c^
	Complete ANS (n = 241)^a^	Partial or no ANS (n = 190)^b^	
Survival at hospital discharge	130/241 (53.9)	69/190 (36.3)	1.70 (1.03-2.79)
Survival at 36 wk PMA	137/241 (56.8)	72/190 (37.9)	1.83 (1.11-3.00)
Survival at 36 wk PMA without major neonatal morbidities^d^	64/238 (26.9)	21/188 (11.2)	2.22 (1.16-4.25)
Severe ICH	63/199 (31.7)	60/120 (50.0)	0.44 (0.24-0.80)
Severe ICH or death^e^	135/236 (57.2)	146/187 (78.1)	0.43 (0.25-0.75)
CPVL	11/199 (5.5)	11/120 (9.2)	0.50 (0.18-1.41)
CPVL or death^e^	112/236 (47.5)	126/187 (67.4)	0.51 (0.30-0.85)
Grade 3 BPD	22/130 (16.9)	14/70 (20.0)	1.02 (0.42-2.46)
Grade 3 BPD or death^e^	126/234 (53.8)	132/188 (70.2)	0.63 (0.38-1.05)
Severe ROP needing treatment	41/140 (29.3)	26/72 (36.1)	0.72 (0.31-1.68)
Severe ROP needing treatment or death^e^	148/239 (61.9)	146/188 (77.7)	0.58 (0.33-1.02)
NEC	26/212 (12.3)	11/133 (8.3)	1.30 (0.56-3.04)
NEC or death^e^	124/237 (52.3)	124/187 (66.3)	0.69 (0.42-1.15)
Surgical NEC	14/212 (6.6)	4/133 (3.0)	2.05 (0.57-7.39)
Surgical NEC or death^e^	113/237 (47.7)	119/187 (63.6)	0.64 (0.39-1.06)
Patent ductus arteriosus	139/214 (65.0)	73/133 (54.9)	1.34 (0.75-2.38)
PDA or death^e^	206/239 (86.2)	170/188 (90.4)	0.66 (0.31-1.42)
Sepsis	100/199 (50.3)	48/114 (42.1)	1.71 (0.98-2.98)
Sepsis or death^e^	160/237 (67.5)	146/187 (78.1)	0.72 (0.41-1.25)
Late-onset sepsis	92/197 (46.7)	43/112 (38.4)	1.68 (0.96-2.94)
Late-onset sepsis or death^e^	156/237 (65.8)	145/187 (77.5)	0.68 (0.39-1.18)

Abbreviations: ANS, antenatal steroids; BPD, bronchopulmonary dysplasia; CPVL, cystic periventricular leukomalacia; GA, gestational age; ICH, intracranial hemorrhage; NEC, necrotizing enterocolitis; OR, odds ratio; PDA, patent ductus arteriosus; PMA, postmenstrual age; ROP, retinopathy of prematurity.

^a^ Complete course of ANS was defined as 2 doses of betamethasone at GA 22 6/7 or earlier.

^b^ Partial course of ANS was defined as 1 dose of betamethasone at GA 22 6/7 or earlier.

^c^ Model adjusted for GA, sex, race, maternal education, small for GA, mode of delivery, multiple birth, prolonged rupture of membranes, year and center of birth as a random effect. Models for CPVL, severe BPD, NEC, sepsis, late-onset sepsis, and surgical NEC could not adjust for center of birth due to small sample size.

^d^ Presence of either severe ICH, cystic PVL, severe BPD, surgical NEC, or severe ROP requiring treatment.

^e^ Composite outcomes include death before 36 weeks’ GA for BPD, NEC, PDA, sepsis, late-onset sepsis and IVH or PVL, and death before discharge for severe ROP.

## Discussion

This cohort study among infants born between GA 22 0/7 and 23 6/7 weeks who received intensive care found that exposure to a complete course of antenatal steroids at GA 22 6/7 weeks or less was independently associated with higher survival, higher survival without major neonatal morbidity, and lower risk of severe ICH, severe ICH or death, CPVL or death, and severe ROP or death. When looking at individual morbidities, the adjusted odds ratio of sepsis was higher in infants born after complete antenatal steroid exposure compared with those with partial antenatal steroid exposure. This was potentially due to the higher survival rate among infants born after exposure to a complete course of antenatal steroids; there was no difference in the composite outcome of sepsis or death.

In a large retrospective study using data from the NICHD NRN, for extremely preterm neonates born between January 1993 and December 2009, Carlo et al^9^ noted a reduction in the combined outcome of NEC or death with the use of antenatal steroids for neonates born at GA 22 weeks (OR, 0.54 [95% CI, 0.30-0.97]). However, there was no difference in the rates of BPD, BPD or death, severe ICH, or severe ICH, CPVL, or death in neonates born at GA 22 weeks among infants with exposure to antenatal steroids compared with those without. A 2018 study by Ehret and colleagues^10^ using data from the Vermont Oxford Network (VON) reported outcomes of extremely preterm infants born at VON member hospitals at GA 22 to 25 weeks between 2012 and 2015. In the study by Ehret et al,^10^ antenatal steroid administration was defined as receipt of steroids at any time prior to delivery. Among infants born at GA 22 weeks, those who were exposed to both antenatal steroids and received postnatal life support were more likely to survive than infants who received postnatal life support only (adjusted risk ratio [aRR], 2.11 [95% CI, 1.68-2.65]). Survival without major neonatal morbidity (ie, BPD, severe ICH, CPVL, sepsis, NEC, or severe ROP) was higher among infants who were exposed to both antenatal steroids and received postnatal life support, compared with infants who received postnatal life support only (aRR, 4.35 [95% CI, 1.84-10.28]).^10^ Like the study by Carlo et al,^9^ Ehret et al^10^ did not report data regarding the GA at exposure to antenatal steroids or the dose or number of courses of antenatal steroids prior to delivery. Neonatal outcomes were assessed based on antenatal steroid exposure at delivery GA and not based on the GA at the time of receipt of antenatal steroids.

In another large cohort study using data from National Center for Health Statistics, Rossi and colleagues^19^ compared survival to age 1 year for extremely preterm infants born at GA 22 weeks with vs without exposure to antenatal steroids. Among infants born at GA 22 weeks, those who were exposed to both antenatal steroids and received postnatal life support were more likely to survive to age 1 year than infants who received postnatal life support only (aRR, 1.6 [95% CI, 1.2-2.1]).^19^

The data from the our study support the September 2021 ACOG and SMFM recommendation^4^ to consider antenatal steroids at GA 22 weeks when there is an intention for providing postnatal intensive care. Given the magnitude of association and consistency with the prior studies by Carlo et al^9^ and Ehret et al,^10^ our findings provide additional support regarding the provision of antenatal steroids to mothers at 22 weeks’ gestation. The small number of infants who were exposed to antenatal steroids at GA 21 weeks precludes separate evaluation of the safety or efficacy of antenatal steroid exposure at this GA.

Strengths of our study include a recent study period (January 2016 to December 2019) with prospectively collected data using prespecified outcomes. The multicenter design of our study increases the generalizability of study findings. This study provides details regarding the number of doses of antenatal steroids and comparison of outcomes in relation to no, partial, and complete antenatal steroids courses. Previous studies have evaluated the outcomes of neonates born at GA 22 weeks and have excluded many neonates who were exposed to antenatal steroids at GA 22 weeks but delivered at GA 23 weeks. We addressed this knowledge gap by considering gestational age at the time of antenatal steroid exposure.

As of September 2021, the ACOG and the SMFM indicate that antenatal steroids administration is recommended at 24 weeks’ gestation and antenatal steroids administration may be considered at 23 weeks’ gestation (level 2B) and at 22 weeks’ gestation (level 2C) after appropriate counseling and if neonatal resuscitation is planned.^4,20^ However, this guidance is based on low certainty evidence. Due to differences in center practices and possible lack of equipoise for some clinicians, a randomized clinical trial to evaluate the beneficial effects of antenatal steroids for pregnant women at 22 weeks’ gestation would be very challenging and is not likely to occur. The only proposed trial listed on ClinicalTrials.gov was canceled due to lack of equipoise.^12,21^ The information from our study strengthens the evidence base in support of a role for antenatal steroids at GA 22 weeks and supports current recommendations.

### Limitations

This study has some limitations. The observational design of the study only suggests associations with differential exposure to antenatal steroids and does not demonstrate a cause-and-effect relationship. The observational design of the study may result in residual confounding. There is a possibility that unknown differences in baseline characteristics, indications for delivery, or other aspects of clinical care associated with both the decision to provide antenatal steroids and neonatal care may affect the findings. To reduce this limitation, we excluded infants who died without attempts at lifesaving postnatal care (ie, those who received comfort care only). Using logistic regression, we also controlled for many known potential factors associated with neonatal survival and morbidities, including race, sex, GA at birth, mode of delivery, multiple gestation, PROM, SGA status, birth year, and center of birth. Some infants may have been exposed to antenatal steroids at GA 21 to 22 weeks but were not born during the GA window; such infants were not included in this analysis. Due to the large number of statistical comparisons, analyses regarding the associations of antenatal steroids with specific morbidities should be considered exploratory. The analysis for death or CPVL might be unduly influenced by the effect on death. However, a comparison for CPVL alone may also be biased because infants who died could not always be assessed for it or may not have had opportunity to develop it. The point estimate for CPVL (0.41) and CPVL or death (0.45) were in the same direction. We did not have adequate power to evaluate the role of partial course of antenatal steroids separately compared with no antenatal steroids.

## Conclusions

In this cohort study among infants born between GA 22 0/7 and 23 6/7 weeks who received intensive care, exposure to a complete course of antenatal steroids at GA 21 to 22 weeks was independently associated with greater odds of survival and survival without major neonatal morbidity. These results provide additional evidence in support of recent modification of perinatal practices and guidelines and may help in further clarification of these guidelines.

## Notes

Supplement 1. **eFigure.** Neonatal Outcomes of Death, Survival Without Any Morbidity, and Survival With Number of Severe Morbidities Stratified by Antenatal Steroid Exposure

Supplement 2.
The Eunice Kennedy Shriver National Institute of Child Health and Human Development Neonatal Research Network

## References

[1] Roberts D, Brown J, Medley N, Dalziel SR. Antenatal corticosteroids for accelerating fetal lung maturation for women at risk of preterm birth. Cochrane Database Syst Rev. 2017;3:CD004454. doi:10.1002/14651858.CD004454.pub3 28321847 PMC6464568

[2] Liggins GC, Howie RN. A controlled trial of antepartum glucocorticoid treatment for prevention of the respiratory distress syndrome in premature infants. Pediatrics. 1972;50(4):515-525. doi:10.1542/peds.50.4.515 4561295

[3] Crowley P, Chalmers I, Keirse MJ. The effects of corticosteroid administration before preterm delivery: an overview of the evidence from controlled trials. Br J Obstet Gynaecol. 1990;97(1):11-25. doi:10.1111/j.1471-0528.1990.tb01711.x 2137711

[4] Cahill AG, Kaimal AJ, Kuller JA, Turrentine MA; American College of Obstetricians and Gynecologists; Society for Maternal-Fetal Medicine. Use of antenatal corticosteroids at 22 weeks of gestation. Accessed August 24, 2022. https://www.acog.org/clinical/clinical-guidance/practice-advisory/articles/2021/09/use-of-antenatal-corticosteroids-at-22-weeks-of-gestation

[5] Mactier H, Bates SE, Johnston T, ; BAPM Working Group. Perinatal management of extreme preterm birth before 27 weeks of gestation: a framework for practice. Arch Dis Child Fetal Neonatal Ed. 2020;105(3):232-239. doi:10.1136/archdischild-2019-318402 31980443

[6] Chawla S, Natarajan G, Shankaran S, ; National Institute of Child Health and Human Development Neonatal Research Network. Association of neurodevelopmental outcomes and neonatal morbidities of extremely premature infants with differential exposure to antenatal steroids. JAMA Pediatr. 2016;170(12):1164-1172. doi:10.1001/jamapediatrics.2016.1936 27723868 PMC5294968

[7] Deshmukh M, Patole S. Antenatal corticosteroids for neonates born before 25 weeks—a systematic review and meta-analysis. PLoS One. 2017;12(5):e0176090. doi:10.1371/journal.pone.0176090 28486556 PMC5423600

[8] Park CK, Isayama T, McDonald SD. Antenatal corticosteroid therapy before 24 weeks of gestation: a systematic review and meta-analysis. Obstet Gynecol. 2016;127(4):715-725. doi:10.1097/AOG.0000000000001355 26959200

[9] Carlo WA, McDonald SA, Fanaroff AA, ; Eunice Kennedy Shriver National Institute of Child Health and Human Development Neonatal Research Network. Association of antenatal corticosteroids with mortality and neurodevelopmental outcomes among infants born at 22 to 25 weeks’ gestation. JAMA. 2011;306(21):2348-2358. doi:10.1001/jama.2011.1752 22147379 PMC3565238

[10] Ehret DEY, Edwards EM, Greenberg LT, . Association of antenatal steroid exposure with survival among infants receiving postnatal life support at 22 to 25 weeks’ gestation. JAMA Netw Open. 2018;1(6):e183235. doi:10.1001/jamanetworkopen.2018.3235 30646235 PMC6324435

[11] Mori R, Kusuda S, Fujimura M; Neonatal Research Network Japan. Antenatal corticosteroids promote survival of extremely preterm infants born at 22 to 23 weeks of gestation. J Pediatr. 2011;159(1):110-114.e1. doi:10.1016/j.jpeds.2010.12.039 21334006

[12] Rysavy MA, Mehler K, Oberthür A, . An immature science: intensive care for infants born at ≤23 weeks of gestation. J Pediatr. 2021;233:16-25.e1. doi:10.1016/j.jpeds.2021.03.006 33691163 PMC8154715

[13] Brumbaugh JE, Hansen NI, Bell EF, ; National Institute of Child Health and Human Development Neonatal Research Network. Outcomes of extremely preterm infants with birth weight less than 400 g. JAMA Pediatr. 2019;173(5):434-445. doi:10.1001/jamapediatrics.2019.0180 30907941 PMC6503635

[14] Papile LA, Burstein J, Burstein R, Koffler H. Incidence and evolution of subependymal and intraventricular hemorrhage: a study of infants with birth weights less than 1,500 gm. J Pediatr. 1978;92(4):529-534. doi:10.1016/S0022-3476(78)80282-0 305471

[15] Bell MJ, Ternberg JL, Feigin RD, . Neonatal necrotizing enterocolitis: therapeutic decisions based upon clinical staging. Ann Surg. 1978;187(1):1-7. doi:10.1097/00000658-197801000-00001 413500 PMC1396409

[16] Walsh MC, Kliegman RM. Necrotizing enterocolitis: treatment based on staging criteria. Pediatr Clin North Am. 1986;33(1):179-201. doi:10.1016/S0031-3955(16)34975-6 3081865 PMC7131118

[17] Jensen EA, Dysart K, Gantz MG, . The diagnosis of bronchopulmonary dysplasia in very preterm infants. an evidence-based approach. Am J Respir Crit Care Med. 2019;200(6):751-759. doi:10.1164/rccm.201812-2348OC 30995069 PMC6775872

[18] VanderWeele TJ, Ding P. Sensitivity analysis in observational research: introducing the E-value. Ann Intern Med. 2017;167(4):268-274. doi:10.7326/M16-2607 28693043

[19] Rossi RM, DeFranco EA, Hall ES. Association of antenatal corticosteroid exposure and infant survival at 22 and 23 weeks. Am J Perinatol. Published online November 28, 2021. doi:10.1055/s-0041-1740062 34839472

[20] Ecker JKA, Mercer B, Blackwell SO, . Obstetric Care Consensus: Interim Update. The American College of Obstetricians and Gynecologists and Society for Maternal-Fetal Medicine; 2017.

[21] Mednax Center for Research, Education, Quality and Safety. Effectiveness of ACS in extreme preemies. Accessed February 11, 2022. https://clinicaltrials.gov/ct2/show/NCT02351310

