# Supplementary material for: Association of Antenatal Steroid Exposure at 21 to 22 Weeks of Gestation With Neonatal Survival and Survival Without Morbidities
Source: JAMA Netw Open. 2022 Sep 26;5(9):e2233331. doi: 10.1001/jamanetworkopen.2022.33331 (PMC9513645; doi:10.1001/jamanetworkopen.2022.33331)
Supplement: Supplement 1. — eFigure. Neonatal Outcomes of Death, Survival Without Any Morbidity, and Survival With Number of Severe Morbidities Stratified by Antenatal Steroid Exposure [file jamanetwopen-e2233331-s001.pdf]

Supplemental Online Content

Chawla S, Wyckoff MH, Rysavy MA, et al; Eunice Kennedy Shriver National Institute of Child Health and Human Development Neonatal Research Network. Association of antenatal steroid exposure at 21 to 22 weeks of gestation with neonatal survival and survival without morbidities. *JAMA Netw Open*. 2022;5(9):e2233331. doi:10.1001/jamanetworkopen.2022.33331

eFigure. Neonatal Outcomes of Death, Survival Without Any Morbidity, and Survival With Number of Severe Morbidities Stratified by Antenatal Steroid Exposure

This supplemental material has been provided by the authors to give readers additional information about their work.

© 2022 Chawla S et al. *JAMA Network Open.*

eFigure. Neonatal Outcomes of Death, Survival Without Any Morbidity, and Survival With Number of Severe Morbidities Stratified by Antenatal Steroid Exposure

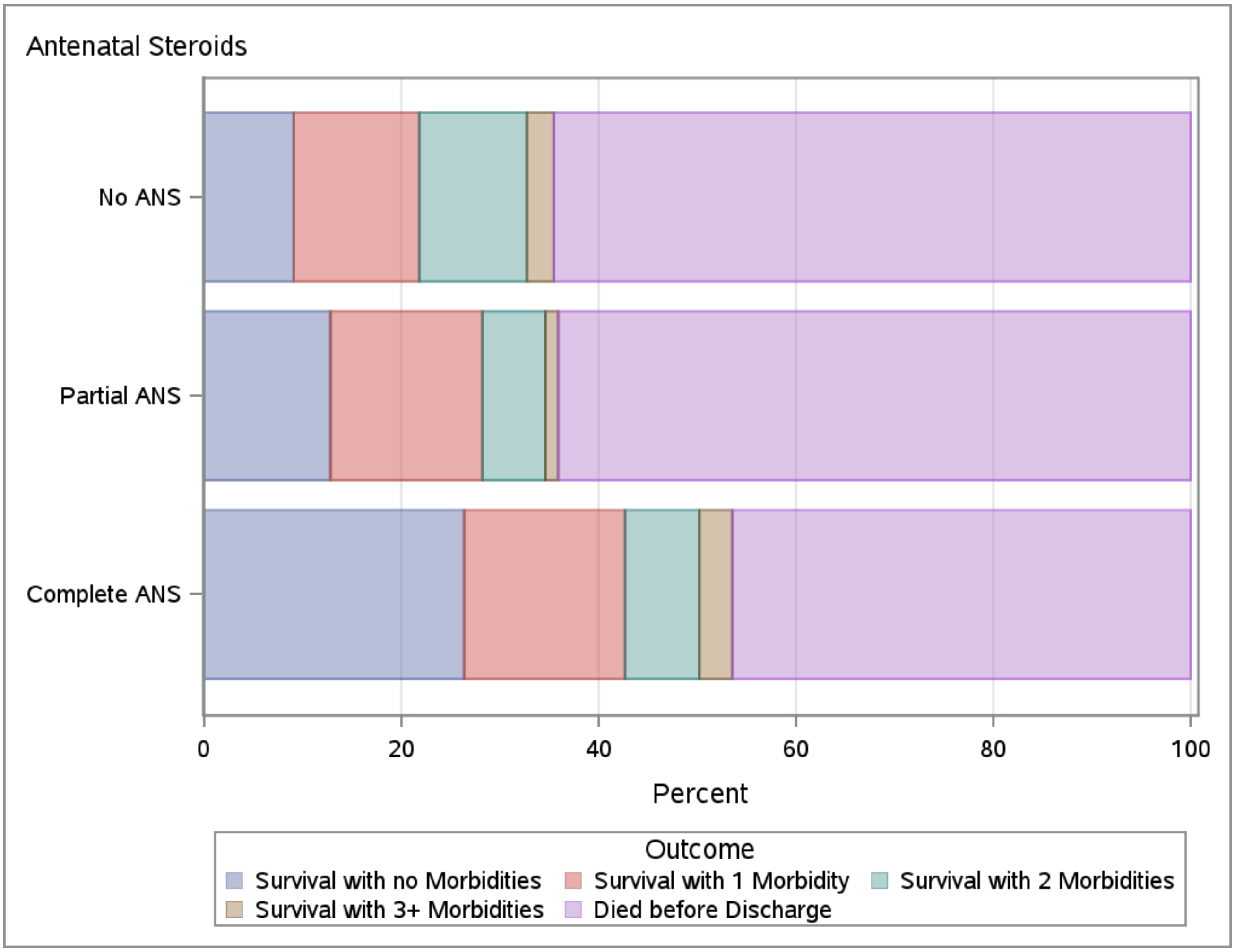

© 2022 Chawla S et al. *JAMA Network Open.*
