# Supplementary material for: Association of Antenatal Steroid Exposure at 21 to 22 Weeks of Gestation With Neonatal Survival and Survival Without Morbidities
Source: JAMA Netw Open. 2022 Sep 26;5(9):e2233331. doi: 10.1001/jamanetworkopen.2022.33331 (PMC9513645; doi:10.1001/jamanetworkopen.2022.33331)
Supplement: Supplement 2. — The Eunice Kennedy Shriver National Institute of Child Health and Human Development Neonatal Research Network [file jamanetwopen-e2233331-s002.pdf]

Supplemental Online Content: Nonauthor Collaborators
*First name, last name, and suffix (if applicable) are required and will appear in PubMed.

*Group Name(s): Eunice Kennedy Shriver National Institute of Child Health and Human Development Neonatal Research Network							
***First Name and Middle Initial(s)**	***Last Name**	***Suffix (eg, Jr, III)**	**Academic Degrees**	**Institution**	**Location (city, state/province, country)**	**Role or Contribution, eg, chair, principal investigator**	**Group (if more than 1 Group listed in the byline) and/or Subgroup (eg, Steering Committee)**
Monica V.	Collins		RN BSN MaEd	Division of Neonatology, University of Alabama at Birmingham	Birmingham, AL, USA	Research Coordinator	
Shirley S.	Cosby		RN BSN	Division of Neonatology, University of Alabama at Birmingham	Birmingham, AL, USA	Research Coordinator	
Angelita M.	Hensman		PhD RNC-NIC	Department of Pediatrics, Women & Infants’ Hospital, Brown University	Providence, RI, USA	Research Coordinator	Generic Database Subcommittee
Martin	Keszler		MD	Department of Pediatrics, Women & Infants’ Hospital, Brown University	Providence, RI, USA	Co-Principal Investigator	
Lucille	St. Pierre		BS	Department of Pediatrics, Women & Infants’ Hospital, Brown University	Providence, RI, USA	Research Assistant	
Elisa	Vieira		RN BSN	Department of Pediatrics, Women & Infants’ Hospital, Brown University	Providence, RI, USA	Research Nurse	
Stephanie	Guilford		BS	Department of Pediatrics, University of Buffalo Women's and Children's Hospital of Buffalo	Buffalo, NY, USA	Research Coordinator	
Emily	Li		BA	Department of Pediatrics, University of Buffalo Women's and Children's Hospital of Buffalo	Buffalo, NY, USA	Research Nurse	
Anne Marie	Reynolds		MD MPH	Department of Pediatrics, University of Buffalo Women's and Children's Hospital of Buffalo	Buffalo, NY, USA	Co-Principal Investigator	

Supplemental Online Content: Nonauthor Collaborators
*First name, last name, and suffix (if applicable) are required and will appear in PubMed.

*First Name and Middle Initial(s)	*Last Name	*Suffix (eg, Jr, III)	Academic Degrees	Institution	Location (city, state/province, country)	Role or Contribution, eg, chair, principal investigator	Group (if more than 1 Group listed in the byline) and/or Subgroup (eg, Steering Committee)
Michael G.	Sacilowski		MAT CCRC	Department of Pediatrics, University of Buffalo Women's and Children's Hospital of Buffalo	Buffalo, NY, USA	Follow-up Coordinator	
Anna Maria	Hibbs		MD MSCE	Department of Pediatrics, Rainbow Babies & Children’s Hospital, Case Western Reserve University	Cleveland, OH, USA	Co-Principal Investigator	Steering Committee; Generic Database Subcommittee
Nancy S.	Newman		RN	Department of Pediatrics, Rainbow Babies & Children’s Hospital, Case Western Reserve University	Cleveland, OH, USA	Research Coordinator	Generic Database Subcommittee
Bonnie S.	Siner		RN	Department of Pediatrics, Rainbow Babies & Children’s Hospital, Case Western Reserve University	Cleveland, OH, USA	Follow-up Coordinator	
Michele C.	Walsh		MD MS	Department of Pediatrics, Rainbow Babies & Children’s Hospital, Case Western Reserve University	Cleveland, OH, USA	Principal Investigator	
Angelia	Williams			Department of Pediatrics, Rainbow Babies & Children’s Hospital, Case Western Reserve University	Cleveland, OH, USA	Research Assistant	
Traci	Beiersdorfer		RN BSN	Cincinnati Children’s Hospital Medical Center, Department of Pediatrics, University of Cincinnati College of Medicine	Cincinnati, OH, USA	Research Nurse	
Cathy	Grisby		BSN CCRC	Cincinnati Children’s Hospital Medical Center, Department of Pediatrics, University of Cincinnati College of Medicine	Cincinnati, OH, USA	Research Coordinator	

Supplemental Online Content: Nonauthor Collaborators
*First name, last name, and suffix (if applicable) are required and will appear in PubMed.

*First Name and Middle Initial(s)	*Last Name	*Suffix (eg, Jr, III)	Academic Degrees	Institution	Location (city, state/province, country)	Role or Contribution, eg, chair, principal investigator	Group (if more than 1 Group listed in the byline) and/or Subgroup (eg, Steering Committee)
Kristin	Kirker		CRC	Cincinnati Children's Hospital Medical Center, Department of Pediatrics, University of Cincinnati College of Medicine	Cincinnati, OH, USA	Research Nurse	
Brenda B.	Poindexter		MD MS	Cincinnati Children's Hospital Medical Center, Department of Pediatrics, University of Cincinnati College of Medicine	Cincinnati, OH, USA	Co-Principal Investigator	Steering Committee
Kurt	Schibler		MD	Cincinnati Children's Hospital Medical Center, Department of Pediatrics, University of Cincinnati College of Medicine	Cincinnati, OH, USA	Principal Investigator	
Julia	Thompson		RN BSN	Cincinnati Children's Hospital Medical Center, Department of Pediatrics, University of Cincinnati College of Medicine	Cincinnati, OH, USA	Research Nurse	
Richard A.	Polin		MD	Division of Neonatology, College of Physicians and Surgeons, Columbia University, New York, NY	New York, NY, USA	Steering Committee Chair	Steering Committee
Luc P.	Brion		MD	Department of Pediatrics, University of Texas Southwestern Medical Center	Dallas, TX, USA	Co-Principal Investigator	
Maria M.	De Leon		RN BSN	Department of Pediatrics, University of Texas Southwestern Medical Center	Dallas, TX, USA	Research Nurse	
Frances	Eubanks		RN BSN	Department of Pediatrics, University of Texas Southwestern Medical Center	Dallas, TX, USA	Research Nurse	

Supplemental Online Content: Nonauthor Collaborators
*First name, last name, and suffix (if applicable) are required and will appear in PubMed.

*First Name and Middle Initial(s)	*Last Name	*Suffix (eg, Jr, III)	Academic Degrees	Institution	Location (city, state/province, country)	Role or Contribution, eg, chair, principal investigator	Group (if more than 1 Group listed in the byline) and/or Subgroup (eg, Steering Committee)
Pollieanna	Sepulveda		RN BSN	Department of Pediatrics, University of Texas Southwestern Medical Center	Dallas, TX, USA	Research Nurse	
Diana M.	Vasil		MSN RNC-NIC BSN	Department of Pediatrics, University of Texas Southwestern Medical Center	Dallas, TX, USA	Research Coordinator	
C. Michael	Cotten		MD MHS	Department of Pediatrics, Duke University	Durham, NC, USA	Principal Investigator	Steering Committee
Joanne	Finkle		RN JD	Department of Pediatrics, Duke University	Durham, NC, USA	Research Coordinator	
Kimberley A.	Fisher		PhD FNP-BC IBCLC	Department of Pediatrics, Duke University	Durham, NC, USA	Research Coordinator	
Ronald N.	Goldberg		MD	Department of Pediatrics, Duke University	Durham, NC, USA	Co-Principal Investigator	
Kelly	Bear		MD	Department of Pediatrics, East Carolina University	Greenville, NC, USA	Site Investigator	
Vicki	Bergstedt		RN	Department of Pediatrics, East Carolina University	Greenville, NC, USA	Research Nurse	
Ryan	Moore		MD	Department of Pediatrics, East Carolina University	Greenville, NC, USA	Site Investigator	
Sherry	Moseley		RN	Department of Pediatrics, East Carolina University	Greenville, NC, USA	Research Coordinator	
Diane I.	Bottcher		RN MSN	Department of Pediatrics, Emory University School of Medicine and Children's Healthcare of Atlanta	Atlanta, GA, USA	Research Nurse	
David P.	Carlton		MD	Department of Pediatrics, Emory University School of Medicine and Children's Healthcare of Atlanta	Atlanta, GA, USA	Principal Investigator	
Yvonne C.	Loggins		RN BSN	Department of Pediatrics, Emory University School of Medicine and Children's Healthcare of Atlanta	Atlanta, GA, USA	Research Coordinator	

*First name, last name, and suffix (if applicable) are required and will appear in PubMed.

*First Name and Middle Initial(s)	*Last Name	*Suffix (eg, Jr, III)	Academic Degrees	Institution	Location (city, state/province, country)	Role or Contribution, eg, chair, principal investigator	Group (if more than 1 Group listed in the byline) and/or Subgroup (eg, Steering Committee)
Colleen	Mackie		BS RT	Department of Pediatrics, Emory University School of Medicine and Children's Healthcare of Atlanta	Atlanta, GA, USA	Research Nurse	
Claudia I.	Franco		RNC MSN	Department of Pediatrics, McGovern Medical School at The University of Texas Health Science Center at Houston	Houston, TX, USA	Research Coordinator	
Kathleen A.	Kennedy		MD MPH	Department of Pediatrics, McGovern Medical School at The University of Texas Health Science Center at Houston	Houston, TX, USA	Principal Investigator	
Amir M.	Khan		MD	Department of Pediatrics, McGovern Medical School at The University of Texas Health Science Center at Houston	Houston, TX, USA	Co-Principal Investigator	
Anna E.	Lis		RN BSN	Department of Pediatrics, McGovern Medical School at The University of Texas Health Science Center at Houston	Houston, TX, USA	Research Coordinator	
Sara C.	Martin		RN BSN	Department of Pediatrics, McGovern Medical School at The University of Texas Health Science Center at Houston	Houston, TX, USA	Research Nurse	
Georgia Elaine	McDavid		RN	Department of Pediatrics, McGovern Medical School at The University of Texas Health Science Center at Houston	Houston, TX, USA	Research Coordinator	
Patricia Ann	Orekoya		RN BSN	Department of Pediatrics, McGovern Medical School at The University of Texas Health Science Center at Houston	Houston, TX, USA	Research Coordinator	

Supplemental Online Content: Nonauthor Collaborators
*First name, last name, and suffix (if applicable) are required and will appear in PubMed.

*First Name and Middle Initial(s)	*Last Name	*Suffix (eg, Jr, III)	Academic Degrees	Institution	Location (city, state/province, country)	Role or Contribution, eg, chair, principal investigator	Group (if more than 1 Group listed in the byline) and/or Subgroup (eg, Steering Committee)
Claudia	Pedroza		PhD	Department of Pediatrics, McGovern Medical School at The University of Texas Health Science Center at Houston	Houston, TX, USA	Statistician	
Patti L.	Pierce Tate		RCP	Department of Pediatrics, McGovern Medical School at The University of Texas Health Science Center at Houston	Houston, TX, USA	Research Nurse	
Emily K.	Stephens		BSN RNC-NIC	Department of Pediatrics, McGovern Medical School at The University of Texas Health Science Center at Houston	Houston, TX, USA	Research Coordinator	
Jon E.	Tyson		MD MPH	Department of Pediatrics, McGovern Medical School at The University of Texas Health Science Center at Houston	Houston, TX, USA	Principal Investigator	Steering Committee
Susan	Gunn		NNP-BC CCRC	Department of Pediatrics, Indiana University School of Medicine	Indianapolis, IN, USA	Research Nurse	
Dianne E.	Herron		RN CCRC	Department of Pediatrics, Indiana University School of Medicine	Indianapolis, IN, USA	Research Coordinator	
Jeffery	Joyce		CCRC (deceased)	Department of Pediatrics, Indiana University School of Medicine	Indianapolis, IN, USA	Research Coordinator	
Gregory M.	Sokol		MD	Department of Pediatrics, Indiana University School of Medicine	Indianapolis, IN, USA	Principal Investigator	
Tarah T.	Colaizy		MD MPH	Department of Pediatrics, University of Iowa	Iowa City, IA, USA	Co-Principal Investigator	
Sarah E.	Faruqui		MSN RN	Department of Pediatrics, University of Iowa	Iowa City, IA, USA	Research Nurse	
Claire A.	Goeke		RN	Department of Pediatrics, University of Iowa	Iowa City, IA, USA	Research Nurse	

Supplemental Online Content: Nonauthor Collaborators
*First name, last name, and suffix (if applicable) are required and will appear in PubMed.

*First Name and Middle Initial(s)	*Last Name	*Suffix (eg, Jr, III)	Academic Degrees	Institution	Location (city, state/province, country)	Role or Contribution, eg, chair, principal investigator	Group (if more than 1 Group listed in the byline) and/or Subgroup (eg, Steering Committee)
Karen J.	Johnson		RN BSN	Department of Pediatrics, University of Iowa	Iowa City, IA, USA	Research Coordinator	
Mendi L.	Schmelzel		MSN RN	Department of Pediatrics, University of Iowa	Iowa City, IA, USA	Research Nurse	
Jacky R.	Walker		RN	Department of Pediatrics, University of Iowa	Iowa City, IA, USA	Research Nurse	
Lisa	Gaetano		RN MSN	Department of Pediatrics, Children's Mercy Hospital	Kansas City, MO, USA	Research Nurse	
Cheri	Gauldin		RN BSN CCRC	Department of Pediatrics, Children's Mercy Hospital	Kansas City, MO, USA	Research Coordinator	
Anne M.	Holmes		RN MSN MBA-HCM CCRC	Department of Pediatrics, Children's Mercy Hospital	Kansas City, MO, USA	Research Nurse	
Howard W.	Kilbride		MD	Department of Pediatrics, Children's Mercy Hospital	Kansas City, MO, USA	Follow-up Principal Investigator	
Eugenia K.	Pallotto		MD MSCE	Department of Pediatrics, University of Missouri School of Medicine	Kansas City, MO, USA	Site Investigator	
Prabhu S.	Parimi		MD	Department of Pediatrics, University of Missouri School of Medicine	Kansas City, MO, USA	Site Investigator	
Allison	Scott		RNC-NIC BSN CCRC	Department of Pediatrics, Children's Mercy Hospital	Kansas City, MO, USA	Research Coordinator	
William E.	Truog		MD	Department of Pediatrics, Children's Mercy Hospital	Kansas City, MO, USA	Principal Investigator	
Erna	Clark		BA	Department of Pediatrics, Nationwide Children’s Hospital, The Ohio State University College of Medicine	Columbus, OH, USA	Research Assistant	

Supplemental Online Content: Nonauthor Collaborators
*First name, last name, and suffix (if applicable) are required and will appear in PubMed.

*First Name and Middle Initial(s)	*Last Name	*Suffix (eg, Jr, III)	Academic Degrees	Institution	Location (city, state/province, country)	Role or Contribution, eg, chair, principal investigator	Group (if more than 1 Group listed in the byline) and/or Subgroup (eg, Steering Committee)
Julie	Gutentag		RN BSN	Department of Pediatrics, Nationwide Children's Hospital, The Ohio State University College of Medicine	Columbus, OH, USA	Research Nurse	
Sudarshan R.	Jadcherla		MD FRCP (Irel) DCH AGAF	Department of Pediatrics, Nationwide Children's Hospital, The Ohio State University College of Medicine	Columbus, OH, USA	Co-Principal Investigator	
Patricia	Luzader		RN	Department of Pediatrics, Nationwide Children's Hospital, The Ohio State University College of Medicine	Columbus, OH, USA	Research Coordinator	
Leif D.	Nelin		MD	Department of Pediatrics, Nationwide Children's Hospital, The Ohio State University College of Medicine	Columbus, OH, USA	Principal Investigator	
Courtney	Park		RN BSN	Department of Pediatrics, Nationwide Children's Hospital, The Ohio State University College of Medicine	Columbus, OH, USA	Research Nurse	
Pablo J.	Sánchez		MD	Department of Pediatrics, Nationwide Children's Hospital, The Ohio State University College of Medicine	Columbus, OH, USA	Principal Investigator	Steering Committee; `
Julie C.	Shadd		BSN RD	Department of Pediatrics, Nationwide Children's Hospital, The Ohio State University College of Medicine	Columbus, OH, USA	Research Assistant	

Supplemental Online Content: Nonauthor Collaborators
*First name, last name, and suffix (if applicable) are required and will appear in PubMed.

*First Name and Middle Initial(s)	*Last Name	*Suffix (eg, Jr, III)	Academic Degrees	Institution	Location (city, state/province, country)	Role or Contribution, eg, chair, principal investigator	Group (if more than 1 Group listed in the byline) and/or Subgroup (eg, Steering Committee)
Melanie	Stein		RRT BBA	Department of Pediatrics, Nationwide Children's Hospital, The Ohio State University College of Medicine	Columbus, OH, USA	Research Assistant	
Margaret	Sullivan		BS	Department of Pediatrics, Nationwide Children's Hospital, The Ohio State University College of Medicine	Columbus, OH, USA	Research Assistant	
Andrew A.	Bremer		MD PhD	Eunice Kennedy Shriver National Institute of Child Health and Human Development, National Institutes of Health	Bethesda, MD, USA	Program Officer	
Rosemary D.	Higgins		MD	Eunice Kennedy Shriver National Institute of Child Health and Human Development, National Institutes of Health	Bethesda, MD, USA	Project Scientist	Steering Committee
Stephanie	Wilson Archer		MA	Eunice Kennedy Shriver National Institute of Child Health and Human Development, National Institutes of Health	Bethesda, MD, USA	Program Coordinator	
Soraya	Abbasi		MD	Department of Pediatrics, University of Pennsylvania Perelman School of Medicine	Philadelphia, PA, USA	Site Investigator	
Christine	Catts		CRNP	Department of Pediatrics, University of Pennsylvania Perelman School of Medicine	Philadelphia, PA, USA	Research Assistant	
Aasma S.	Chaudhary		BS RRT	Department of Pediatrics, University of Pennsylvania Perelman School of Medicine	Philadelphia, PA, USA	Research Coordinator	

Supplemental Online Content: Nonauthor Collaborators

*First name, last name, and suffix (if applicable) are required and will appear in PubMed.

*First Name and Middle Initial(s)	*Last Name	*Suffix (eg, Jr, III)	Academic Degrees	Institution	Location (city, state/province, country)	Role or Contribution, eg, chair, principal investigator	Group (if more than 1 Group listed in the byline) and/or Subgroup (eg, Steering Committee)
Sara B.	DeMauro		MD MSCE	Department of Pediatrics, University of Pennsylvania Perelman School of Medicine	Philadelphia, PA, USA	Principal Investigator	Steering Committee
Megan A.	Dhawan		MSN CRNP	Department of Pediatrics, University of Pennsylvania Perelman School of Medicine	Philadelphia, PA, USA	Research Nurse	
Eric C.	Eichenwald		MD	Department of Pediatrics, University of Pennsylvania Perelman School of Medicine	Philadelphia, PA, USA	Co-Principal Investigator	Generic Database Subcommittee
Sarvin	Ghavam		MD	Department of Pediatrics, University of Pennsylvania Perelman School of Medicine	Philadelphia, PA, USA	Site Investigator	
Haresh	Kirpalani		BM MSc	Department of Pediatrics, University of Pennsylvania Perelman School of Medicine	Philadelphia, PA, USA	Co-Principal Investigator	
Toni	Mancini		RN BSN CCRC	Department of Pediatrics, University of Pennsylvania Perelman School of Medicine	Philadelphia, PA, USA	Research Coordinator	
Barbara	Schmidt		MD MSc	Department of Pediatrics, University of Pennsylvania Perelman School of Medicine	Philadelphia, PA, USA	Principal Investigator	
Jonathan M.	Snyder		RN BSN	Department of Pediatrics, University of Pennsylvania Perelman School of Medicine	Philadelphia, PA, USA	Research Nurse	
Kyle	Binion		BS	University of Rochester School of Medicine and Dentistry	Rochester, NY, USA	Research Assistant	
Elizabeth	Boylin		BA	University of Rochester School of Medicine and Dentistry	Rochester, NY, USA	Research Assistant	
Carl T.	D'Angio		MD	University of Rochester School of Medicine and Dentistry	Rochester, NY, USA	Principal Investigator	Steering Committee

Supplemental Online Content: Nonauthor Collaborators
*First name, last name, and suffix (if applicable) are required and will appear in PubMed.

*First Name and Middle Initial(s)	*Last Name	*Suffix (eg, Jr, III)	Academic Degrees	Institution	Location (city, state/province, country)	Role or Contribution, eg, chair, principal investigator	Group (if more than 1 Group listed in the byline) and/or Subgroup (eg, Steering Committee)
Ronnie	Guillet		MD PhD	University of Rochester School of Medicine and Dentistry	Rochester, NY, USA	Co-Principal Investigator	
Rosemary L.	Jensen			University of Rochester School of Medicine and Dentistry	Rochester, NY, USA	Follow-up Coordinator	
Rachel	Jones			University of Rochester School of Medicine and Dentistry	Rochester, NY, USA	Research Assistant	
Jennifer	Kachelmeyer		BS	University of Rochester School of Medicine and Dentistry	Rochester, NY, USA	Research Assistant	
Alison	Kent		BMBS FRACP MD	University of Rochester School of Medicine and Dentistry	Rochester, NY, USA	Site Investigator	
Deanna	Maffett		RN	University of Rochester School of Medicine and Dentistry	Rochester, NY, USA	Research Nurse	
Constance	Orme		BA	University of Rochester School of Medicine and Dentistry	Rochester, NY, USA	Research Assistant	
Diane M.	Prinzing		AAS	University of Rochester School of Medicine and Dentistry	Rochester, NY, USA	Research Nurse	
Daisy	Rochez		BS MHA	University of Rochester School of Medicine and Dentistry	Rochester, NY, USA	Research Assistant	
Mary	Rowan		RN	University of Rochester School of Medicine and Dentistry	Rochester, NY, USA	Research Nurse	
Premini	Sabaratnam		MPH	University of Rochester School of Medicine and Dentistry	Rochester, NY, USA	Research Assistant	
Ann Marie	Scorsone		MS CCRC	University of Rochester School of Medicine and Dentistry	Rochester, NY, USA	Research Coordinator	
Holly I.M.	Wadkins		MA	University of Rochester School of Medicine and Dentistry	Rochester, NY, USA	Research Coordinator	
Carla M.	Bann		PhD	Social, Statistical and Environmental Sciences Unit, RTI International	Research Triangle Park, NC, USA	Statistician	

Supplemental Online Content: Nonauthor Collaborators
*First name, last name, and suffix (if applicable) are required and will appear in PubMed.

*First Name and Middle Initial(s)	*Last Name	*Suffix (eg, Jr, III)	Academic Degrees	Institution	Location (city, state/province, country)	Role or Contribution, eg, chair, principal investigator	Group (if more than 1 Group listed in the byline) and/or Subgroup (eg, Steering Committee)
Jenna	Gabrio		MPH CCRP	Social, Statistical and Environmental Sciences Unit, RTI International	Research Triangle Park, NC, USA	Research Coordinator	
Marie G.	Gantz		PhD	Social, Statistical and Environmental Sciences Unit, RTI International	Research Triangle Park, NC, USA	Statistician	
David	Leblond		BS	Social, Statistical and Environmental Sciences Unit, RTI International	Research Triangle Park, NC, USA	Database Programmer	
Jeanette	O'Donnell Auman		BS	Social, Statistical and Environmental Sciences Unit, RTI International	Research Triangle Park, NC, USA	Database Programmer	
Dennis	Wallace		PhD	Social, Statistical and Environmental Sciences Unit, RTI International	Research Triangle Park, NC, USA	Co-Principal Investigator	
Kristin M.	Zaterka-Baxter		RN BSN CCRP	Social, Statistical and Environmental Sciences Unit, RTI International	Research Triangle Park, NC, USA	Research Coordinator	
Michelle L.	Baack		MD	Department of Pediatrics, Sanford School of Medicine-University of South Dakota	Sioux Falls, SD	Site Investigator	
Megan	Broadbent		RN BSN	Department of Pediatrics, Sanford School of Medicine-University of South Dakota	Sioux Falls, SD	Research Nurse	
Chelsey	Elenkiwich		RN BSN	Department of Pediatrics, Sanford School of Medicine-University of South Dakota	Sioux Falls, SD	Research Nurse	
Megan M.	Henning		RN	Department of Pediatrics, Sanford School of Medicine-University of South Dakota	Sioux Falls, SD	Research Nurse	

Supplemental Online Content: Nonauthor Collaborators
*First name, last name, and suffix (if applicable) are required and will appear in PubMed.

*First Name and Middle Initial(s)	*Last Name	*Suffix (eg, Jr, III)	Academic Degrees	Institution	Location (city, state/province, country)	Role or Contribution, eg, chair, principal investigator	Group (if more than 1 Group listed in the byline) and/or Subgroup (eg, Steering Committee)
Sarah	Van Muyden		RN BSN	Department of Pediatrics, Sanford School of Medicine-University of South Dakota	Sioux Falls, SD	Research Nurse	
M. Bethany	Ball		BS CCRC	Department of Pediatrics, Division of Neonatal and Developmental Medicine, Stanford University	Palo Alto, CA, USA	Research Coordinator	Generic Database Subcommittee
Valerie Y.	Chock		MD MS Epi	Department of Pediatrics, Division of Neonatal and Developmental Medicine, Stanford University School of Medicine and Lucile Packard Children's Hospital	Palo Alto, CA, USA	Co-Principal Investigator	
Melinda S.	Proud		RCP	Department of Pediatrics, Division of Neonatal and Developmental Medicine, Stanford University School of Medicine and Lucile Packard Children's Hospital	Palo Alto, CA, USA	Research Assistant	
Elizabeth N.	Reichert		MA CCRC	Department of Pediatrics, Division of Neonatal and Developmental Medicine, Stanford University School of Medicine and Lucile Packard Children's Hospital	Palo Alto, CA, USA	Research Assistant	
Dharshi	Sivakumar		MD	Department of Pediatrics, Division of Neonatal and Developmental Medicine, Stanford University School of Medicine and Lucile Packard Children's Hospital	Palo Alto, CA, USA	Follow-up Examiner	
David K.	Stevenson		MD	Department of Pediatrics, Division of Neonatal and Developmental Medicine, Stanford University School of Medicine and Lucile Packard Children's Hospital	Palo Alto, CA, USA	Co-Principal Investigator	

Supplemental Online Content: Nonauthor Collaborators
*First name, last name, and suffix (if applicable) are required and will appear in PubMed.

*First Name and Middle Initial(s)	*Last Name	*Suffix (eg, Jr, III)	Academic Degrees	Institution	Location (city, state/province, country)	Role or Contribution, eg, chair, principal investigator	Group (if more than 1 Group listed in the byline) and/or Subgroup (eg, Steering Committee)
R. Jordan	Williams		MD	Department of Pediatrics, Division of Neonatal and Developmental Medicine, Stanford University School of Medicine and Lucile Packard Children's Hospital	Palo Alto, CA, USA	Research Assistant	
Teresa	Chanlaw		MPH	Department of Pediatrics, University of California	Los Angeles, CA, USA	Research Coordinator	
Uday	Devaskar		MD	Department of Pediatrics, University of California	Los Angeles, CA, USA	Principal Investigator	
Meena	Garg		MD	Department of Pediatrics, University of California	Los Angeles, CA, USA	Co-Principal Investigator	
Rachel	Geller		RN BSN	Department of Pediatrics, University of California	Los Angeles, CA, USA	Research Coordinator	
Janice	Bernhardt		MS RN	Division of Neonatal/Perinatal Medicine, Department of Pediatrics, University of North Carolina	Chapel Hill, NC, USA	Research Coordinator	
Carl L.	Bose		MD	Division of Neonatal/Perinatal Medicine, Department of Pediatrics, University of North Carolina	Chapel Hill, NC, USA	Site Investigator	
Cynthia L.	Clark		RN	Division of Neonatal/Perinatal Medicine, Department of Pediatrics, University of North Carolina	Chapel Hill, NC, USA	Research Coordinator	
Matthew Maxwell	Laughon		MD MPH	Division of Neonatal/Perinatal Medicine, Department of Pediatrics, University of North Carolina	Chapel Hill, NC, USA	Site Investigator	

Supplemental Online Content: Nonauthor Collaborators
*First name, last name, and suffix (if applicable) are required and will appear in PubMed.

*First Name and Middle Initial(s)	*Last Name	*Suffix (eg, Jr, III)	Academic Degrees	Institution	Location (city, state/province, country)	Role or Contribution, eg, chair, principal investigator	Group (if more than 1 Group listed in the byline) and/or Subgroup (eg, Steering Committee)
Jennifer	Talbert		MS RN BSN RDH	Division of Neonatal/Perinatal Medicine, Department of Pediatrics, University of North Carolina	Chapel Hill, NC, USA	Research Nurse	
Conra	Backstrom Lacy		RN	University of New Mexico Health Sciences Center	Albuquerque, NM, USA	Research Coordinator	
Janell	Fuller		MD	University of New Mexico Health Sciences Center	Albuquerque, NM, USA	Co-Principal Investigator	
Mary	Hanson		RN BSN	University of New Mexico Health Sciences Center	Albuquerque, NM, USA	Research Nurse	
Elizabeth	Kuan		RN BSN	University of New Mexico Health Sciences Center	Albuquerque, NM, USA	Research Nurse	
Robin K.	Ohls		MD	University of New Mexico Health Sciences Center	Albuquerque, NM, USA	Co-Principal Investigator	
Sandra	Sundquist Beauman		MSN RNC-NIC	University of New Mexico Health Sciences Center	Albuquerque, NM, USA	Research Coordinator	
Kristi L.	Watterberg		MD	University of New Mexico Health Sciences Center	Albuquerque, NM, USA	Principal Investigator	Steering Committee
John	Barks		MD	Department of Neonatal-Perinatal Medicine, University of Michigan, C.S. Mott Children's Hospital	Ann Arbor, MI, USA	Site Investigator	
Diane F.	White		RRT CCRP	Department of Neonatal-Perinatal Medicine, University of Michigan, C.S. Mott Children's Hospital	Ann Arbor, MI, USA	Research Assistant	
Mariana	Baserga		MD MSCI	Department of Pediatrics, Division of Neonatology, University of Utah School of Medicine	Salt Lake City, UT, USA	Co-Principal Investigator	
Jill	Burnett		RNC BSN	Department of Pediatrics, Division of Neonatology, University of Utah School of Medicine	Salt Lake City, UT, USA	Research Nurse	

Supplemental Online Content: Nonauthor Collaborators
*First name, last name, and suffix (if applicable) are required and will appear in PubMed.

*First Name and Middle Initial(s)	*Last Name	*Suffix (eg, Jr, III)	Academic Degrees	Institution	Location (city, state/province, country)	Role or Contribution, eg, chair, principal investigator	Group (if more than 1 Group listed in the byline) and/or Subgroup (eg, Steering Committee)
Susan	Christensen		RNC BSN	Department of Pediatrics, Division of Neonatology, University of Utah School of Medicine	Salt Lake City, UT, USA	Research Nurse	
Kathleen	Coleman		RN	Department of Pediatrics, Division of Neonatology, University of Utah School of Medicine	Salt Lake City, UT, USA	Research Nurse	
Brandy	Davis		RN BSN	Department of Pediatrics, Division of Neonatology, University of Utah School of Medicine	Salt Lake City, UT, USA	Research Nurse	
Jennifer O.	Elmont		RN BSN	Department of Pediatrics, Division of Neonatology, University of Utah School of Medicine	Salt Lake City, UT, USA	Research Nurse	
Barbara L.	Francom		RN BSN	Department of Pediatrics, Division of Neonatology, University of Utah School of Medicine	Salt Lake City, UT, USA	Research Nurse	
Jamie	Jordan		RN BSN	Department of Pediatrics, Division of Neonatology, University of Utah School of Medicine	Salt Lake City, UT, USA	Research Nurse	
Manndi C.	Loertscher		BS CCRP	Department of Pediatrics, Division of Neonatology, University of Utah School of Medicine	Salt Lake City, UT, USA	Research Assistant	
Trisha	Marchant		RN BSN	Department of Pediatrics, Division of Neonatology, University of Utah School of Medicine	Salt Lake City, UT, USA	Research Nurse	
Earl	Maxson		BSN	Department of Pediatrics, Division of Neonatology, University of Utah School of Medicine	Salt Lake City, UT, USA	Research Nurse	
Kandace M.	McGrath		BS	Department of Pediatrics, Division of Neonatology, University of Utah School of Medicine	Salt Lake City, UT, USA	Research Assistant	

Supplemental Online Content: Nonauthor Collaborators
*First name, last name, and suffix (if applicable) are required and will appear in PubMed.

*First Name and Middle Initial(s)	*Last Name	*Suffix (eg, Jr, III)	Academic Degrees	Institution	Location (city, state/province, country)	Role or Contribution, eg, chair, principal investigator	Group (if more than 1 Group listed in the byline) and/or Subgroup (eg, Steering Committee)
Hena G.	Mickelsen		BA	Department of Pediatrics, Division of Neonatology, University of Utah School of Medicine	Salt Lake City, UT, USA	Research Assistant	
Stephen D.	Minton		MD	Department of Pediatrics, Division of Neonatology, University of Utah School of Medicine	Salt Lake City, UT, USA	Site Investigator	
D. Melody	Parry		RN BSN	Department of Pediatrics, Division of Neonatology, University of Utah School of Medicine	Salt Lake City, UT, USA	Research Nurse	
Carrie A.	Rau		RN BSN CCRC	Department of Pediatrics, Division of Neonatology, University of Utah School of Medicine	Salt Lake City, UT, USA	Research Coordinator	
Susan T.	Schaefer		RRT RN BSN	Department of Pediatrics, Division of Neonatology, University of Utah School of Medicine	Salt Lake City, UT, USA	Research Nurse	
Mark J.	Sheffield		MD	Department of Pediatrics, Division of Neonatology, University of Utah School of Medicine	Salt Lake City, UT, USA	Site Investigator	
Katherine	Tice		RN BSN	Department of Pediatrics, Division of Neonatology, University of Utah School of Medicine	Salt Lake City, UT, USA	Research Nurse	
Kimberlee	Weaver-Lewis		RN MS	Department of Pediatrics, Division of Neonatology, University of Utah School of Medicine	Salt Lake City, UT, USA	Research Nurse	
Kathryn D.	Woodbury		RN BSN	Department of Pediatrics, Division of Neonatology, University of Utah School of Medicine	Salt Lake City, UT, USA	Research Nurse	
Bradley A.	Yoder		MD	Department of Pediatrics, Division of Neonatology, University of Utah School of Medicine	Salt Lake City, UT, USA	Principal Investigator	Steering Committee

Supplemental Online Content: Nonauthor Collaborators
*First name, last name, and suffix (if applicable) are required and will appear in PubMed.

*First Name and Middle Initial(s)	*Last Name	*Suffix (eg, Jr, III)	Academic Degrees	Institution	Location (city, state/province, country)	Role or Contribution, eg, chair, principal investigator	Group (if more than 1 Group listed in the byline) and/or Subgroup (eg, Steering Committee)
Stephen D.	Kicklighter		MD	Department of Pediatrics, Division of Neonatology, WakeMed Health and Hospitals	Raleigh, NC, USA	Site Investigator	
Ginger	Rhodes-Ryan		ARNP MSN NNP-BC	Department of Pediatrics, Division of Neonatology, WakeMed Health and Hospitals	Raleigh, NC, USA	Research Coordinator	
Donna	White		RN-BC BSN	Department of Pediatrics, Division of Neonatology, WakeMed Health and Hospitals	Raleigh, NC, USA	Research Nurse	
Kirsten	Childs		RN BSN	Department of Pediatrics, Wayne State University	Detroit, MI, USA	Research Nurse	
Bogdan	Panaitescu		MD PhD	Department of Pediatrics, Wayne State University	Detroit, MI, USA	Site Investigator	
